# Estimating the sample size of sham-controlled randomized controlled trials using existing evidence

**DOI:** 10.12688/f1000research.108554.2

**Published:** 2022-11-07

**Authors:** George C.M. Siontis, Adriani Nikolakopoulou, Romy Sweda, Dimitris Mavridis, Georgia Salanti

**Affiliations:** 1 Department of Cardiology, University Hospital of Bern, Bern, Switzerland; 2 Institute of Medical Biometry and Statistics, University of Freiburg, Freiburg, Germany; 3 Department of Primary Education, University of Ioannina, Ioannina, Greece; 4 Institute of Social and Preventive Medicine (ISPM), University of Bern, Bern, Switzerland

**Keywords:** meta-analysis, sequential methods, power calculation, renal sympathetic denervation

## Abstract

**Background:** In randomized controlled trials (RCTs), the power is often ‘reverse engineered’ based on the number of participants that can realistically be achieved. An attractive alternative is planning a new trial conditional on the available evidence; a design of particular interest in RCTs that use a sham control arm (sham-RCTs).

**Methods:** We explore the design of sham-RCTs, the role of sequential meta-analysis and  conditional planning in a systematic review of renal sympathetic denervation for patients with arterial hypertension. The main efficacy endpoint was mean change in 24-hour systolic blood pressure. We performed sequential meta-analysis to identify the time point where the null hypothesis would be rejected in a prospective scenario. Evidence-based conditional sample size calculations were performed based on fixed-effect meta-analysis.

**Results: **In total, six sham-RCTs (981 participants) were identified. The first RCT was considerably larger (535 participants) than those subsequently published (median sample size of 80). All trial sample sizes were calculated assuming an unrealistically large intervention effect which resulted in low power when each study is considered as a stand-alone experiment. Sequential meta-analysis provided firm evidence against the null hypothesis with the synthesis of the first four trials (755 patients, cumulative mean difference -2.75 (95%CI -4.93 to -0.58) favoring the active intervention)). Conditional planning resulted in much larger sample sizes compared to those in the original trials, due to overoptimistic expected effects made by the investigators in individual trials, and potentially a time-effect association.

**Conclusions: **Sequential meta-analysis of sham-RCTs can reach conclusive findings earlier and hence avoid exposing patients to sham-related risks. Conditional planning of new sham-RCTs poses important challenges as many surgical/minimally invasive procedures improve over time, the intervention effect is expected to increase in new studies and this violates the underlying assumptions. Unless this is accounted for, conditional planning will not improve the design of sham-RCTs.

## Introduction

A central decision when designing a randomized-control trial (RCT) is the number of patients that should be enrolled. RCTs including too few participants have been characterized as having limited clinical value and being unethical. ^1^ However, conducting adequately powered trials often presents practical difficulties and investigators sometimes end up performing ‘reverse engineering’ in their sample size calculations. ^2^ Instead of defining the treatment effect that is expected in the particular setting, which along with other parameters will result in the sample size needed, the ‘expected’ treatment effect is derived by the available-based usually on practical and economic considerations-sample size. This practice may result to unrealistically large treatment effects to justify the small number of participants to be enrolled.

Designing a new trial using the existing evidence in the form of meta-analysis has several advantages. ^2^^–^^5^ Meta-analysis provides more powerful and precise effect estimates over individual trials which is an important advantage when the necessary means for conducting a large trial of adequate power are not available. Conditional planning of a new trial means that the calculations for the required sample size are not based on the power of the trial as stand-alone experiment but on the power of the resulting meta-analysis of the available evidence. The concept of conditional planning builds upon and combines ideas of meta-analysis and living systematic reviews, ^6^^,^^7^ which are continuously updated as new data become available over time, and evidence-based sample size calculations ^2^^,^^3^^,^^5^ which base the determination of sample size on the existing available evidence. ^8^ This typically leads to smaller required sample sizes compared to that obtained using the conventional approach. ^9^

Conditional planning of new trials should ideally be placed in a collaborative framework, where investigators of trials on the same topic work together to determine the similarities and differences of their studies and the prospective nature of the meta-analysis. The approach has been recently promoted as a promising route towards expediting drug licensing and inform reimbursement. ^10^

Minimizing the required sample size is particularly important in specific settings where achieving a large sample size in a trial is challenging. This includes interventions for rare diseases, expensive or very cumbersome interventions, early-phase trials in drug development or when the control intervention poses important health risks and raises ethical concerns. RCTs with sham-controlled interventions (sham-RCTs) feature many of these characteristics and are typically small and underpowered. ^11^^–^^13^ This makes the use of conditional planning promising in this context. However, the method makes a series of assumptions. Among others it is assumed that the true underlying effect size (which we assume is unbiasedly estimated by the summary effect) should not change over time. This is rather unlikely to happen in sham-RCTs as the learning curve applies to most surgical/minimally invasive interventions and studies of their efficacy show larger effects over time. Hence, the conditional power approach is both promising and challenging to be applied in this context.

In this paper, we aim to illustrate some of the challenges encountered when sham-RCTs are designed using the conventional approach to calculate the sample size and explore any potential advantages of using existing evidence both in drawing inferences about the differences between the interventions (active versus sham intervention) and when planning a new future study. We included RCTs comparing renal sympathetic denervation to a sham intervention for the control of arterial hypertension in patients with resistant hypertension with or without the combination of different antihypertensive medications. To this aim, we attempt to replicate the sample size calculations as described in individual trials, perform standard and sequential meta-analysis and calculate the sample size that would have been required conditional on the existing evidence in each step of the evidence synthesis.

## Methods

### Systematic review methods

We performed a systematic literature search (last search in December 2019) limited to English-language articles published in Medline and the Cochrane Central Register of Controlled Trials (CENTRAL) using the terms “randomized (randomised) controlled trial”, “sham”, “renal denervation”, “arterial hypertension”, as subject headings and text words, was conducted by one investigator. The detailed search algorithms can be found as *Extended data.* ^43^ The reference lists of original studies, review papers, and relevant meta-analyses of the interventions of interest initially identified by the electronic searches were also reviewed in an attempt to identify additional eligible trials. For each eligible sham-RCT, we also retrieved any publicly available study protocol, in which details related to the study design were provided. We excluded trials which were terminated preterm. No further limitations were applied.

The full text reports of relevant trials and their protocols were retrieved, and data on study design, patient and intervention characteristics, the outcome of interest, time to follow-up, and the exact description of the active intervention were extracted. Information about sample size calculations were independently extracted by two investigators in separate using prespecified data extraction forms. Any discrepancies were resolved by consensus after consulting a third investigator.

We extracted from each study the following information about sample size calculations: type I error, type II error or power, assumptions in the control group (standard deviation), the superiority margin (when relevant) for the primary efficacy endpoint, the anticipated treatment effect (mean difference), the recruitment period, randomization ratio, the calculated sample size and the achieved sample size for each arm. Details related to power calculations were retrieved from the main document, supplementary material, and previously published protocols of the trials. The outcome of interest was mean change in 24-hour ambulatory systolic blood pressure (SBP).

### Sample size recalculations

We first attempted to replicate the sample size calculations described in individual trials. We hypothesized that the tests were two-sided and the type I error at 5% and power 80% unless different assumptions were stated. All other parameters for the power calculations were adopted as reported in the original articles. Sample size recalculations were performed by using the *power* command in Stata 15. ^14^ We also calculated the relative difference between the achieved and initially calculated sample size as (achieved sample size - calculated sample size)/achieved sample size.

### Standard and sequential meta-analysis

We performed standard and sequential pairwise meta-analysis for mean differences (MD). ^15^^,^^16^ We intended to perform random-effects meta-analysis, but as between-study variance (τ ^2^) was estimated at 0 in this setting, our calculations are identical to those from a fixed effect meta-analysis. Meta-analyses of medical interventions may result in false positive or false negative results, due to low statistical power when the required number of randomised participants or trials has not been reached. Under this scenario, trial sequential analysis of a meta-analysis may amend these problems by handling a meta-analysis of several RCTs in an analogous manner to interim analysis of a single RCT. The available sham-RCTs were included in the sequential (cumulative) meta-analysis following the chronological order of publication and drawn boundaries calculated using an adaptation of the continuous alpha-spending function. ^16^^,^^17^ Crossing a boundary indicates strong evidence against the null hypothesis of equal means between active and sham procedures. We recorded the timepoint when one of the boundaries is crossed; this is the time point that the addition of a published study to the meta-analysis rejects the null hypothesis. We called this timepoint ‘ *final*’ indicating that beyond this timepoint no further research is needed. We calculated the ‘ *unnecessary*’ sample size as the total sample size of studies published after the final timepoint. All analyses were performed in R (version 4.0.2; R-Project for Statistical Computing) using the package *meta* and self-programmed routines. ^18^

### Conditional planning of trials assuming a prospective meta-analysis

We examine the scenario where the identified studies were *a-priori* planned and aimed to test the null hypothesis that the mean SBP is the same between active invasive and sham intervention. We calculate the conditional power of meta-analysis to estimate the required sample size in several steps of the analysis. In the sample size calculations, the difference in SBP in the new trial is assumed to be sufficiently similar to the ones observed and included in the meta-analysis. We assume absence of time-effect interaction between effect modifiers and time (i.e. the effect size is the same between early and later studies). We consider the sequential order of the trials until the final timepoint. We start with the first published trial, and we calculate the sample size needed for a second trial which, when added to the first trial their synthesis will lead into a rejection of the null hypothesis using the conditional power method. ^2^ Then, we synthesize the data from the first two published trials and we estimate the sample size needed in a third trial using again the conditional power; the difference in SBP in the new trial is assumed to be sufficiently similar the one estimated from the meta-analysis of the first two trials. We continue until the final timepoint. We compare the estimated sample size and the anticipated effect size from the conditional planning approach to those presented in the original papers. Analyses have been performed in Stata 15 using 1,000 simulations. Box 1 summarizes the key aspects in sample size calculations based on the conditional power of a meta-analysis.

Box 1. **Key aspects in sample size calculations based on conditional power.**

**Table:** 

**Assumed quantities**	
**Type I error** (also known as “false positive”)	The error of rejecting a null hypothesis when it is actually true needs to be defined. It refers to the probability of accepting an alternative hypothesis when the results can be attributed just to chance.
**Type II error** (also known as “false negative”)	The error of not rejecting a null hypothesis when the alternative hypothesis is true needs to be defined. It refers to the error of failing to accept an alternative hypothesis when you don't have adequate power. It occurs when we are failing to observe a difference when in truth there is one.
**Assumed effect size**	The effect size to be considered in power calculations for a future trial based on previous experience, or results from previous meta-analysis of existing evidence, or what is considered clinically relevant.
**Key assumptions**	
**Lack of association between effect/effect modifiers and time**	As in conventional meta-analytic approaches, the assumption that the effect sizes of individual trials are independent should be fulfilled for conditional planning of future trial(s). The true underlying effect size (which we assume is unbiasedly estimated by the summary effect) should not be dependent on time. Similarly, any effect modifiers shall not change over time. Any time-dependent changes in effects would distort the sample size calculations.
**Small heterogeneity**	The variability of the true treatment effect across trials should be low. Otherwise, even the planning of huge trials will not result in the anticipated conditional power.

## Results

### Search findings and characteristics of eligible sham-RCTs

In the Online Figure (see *Extended data* ^43^) we summarize details of the study selection process. Overall, six sham-RCTs (with a total of 981 patients) ^19^^–^^27^ comparing renal sympathetic denervation (n=585 patients) to a sham-intervention (n=396 patients) were deemed eligible ( Table 1). Random allocation was 1:1 in 5 trials ^21^^–^^27^ and 2:1 in one ^19^^,^^20^ of the trials giving more weight to patients randomized to the active intervention. Two of the trials ^23^^–^^25^ were not prospectively powered; this is because they were designed as small-scale proof-of-concept trials to minimize exposure of patients to an interventional procedure with not previously documented efficacy (based on the findings of SYMPLICITY HTN-3 trial ^19^^,^^20^). 24-hour ambulatory SBP and daytime ambulatory SBP were the primary endpoints in 4 and 2 trials, respectively. The majority of the trials (5 out of 6) were single-blinded, but outcome assessment was performed in blinded manner in all trials ( Table 1). Follow-up period for reported results ranged from 2 up to 6 months. While the sample size in the first trial ^20^ was relatively large (535 participants), the sample sizes of subsequent individual trials ranged from 69 to 146 with a median of 80 participants.

**Table 1.** Characteristics of sham-RCTs comparing renal denervation to a sham-intervention considered eligible.

Trials	Recruitment period	Year of publication	Random allocation	Blinding/Trial design	Blind outcome evaluation	Follow up (months)	Funding source	Active arm	Primary endpoint (mean change)
SYMPLICITY HTN-3 ^19^^,^^20^	Oct 2011 to May 2013	2014	2:1	Single-blind/superiority	yes	6	Industry-related	RDN	24-hour ambulatory SBP *
Desch S., *et al*. ^21^	Jul 2012 to Jan 2014	2015	1:1	Single-blind/superiority	yes	6	Non-industry related	RDN	24-hour ambulatory SBP
ReSET ^22^	nd	2016	1:1	Double-blind/not specified	yes	3	Non-industry related	RDN	Daytime ambulatory SBP
SPYRAL HTN-OFF MED ^23^^,^^24^	Jun 2015 to Jan 2017	2017	1:1	Single-blind/na **	yes	3	Industry-related	RDN	24-hour ambulatory SBP
SPYRAL HTN-ON MED ^23^^,^^25^	Jul 2015 to Jun 2017	2018	1:1	Single-blind/na **	yes	6	Industry-related	RDN	24-hour ambulatory SBP
RADIANCE-HTN SOLO ^26^^,^^27^	Mar 2016 to Dec 2017	2018	1:1	Single-blind/superiority	yes	2 and 6	Industry-related	Ultrasound renal denervation	Daytime ambulatory SBP

Abbreviations: RCT, randomized controlled trial; RND, radiofrequency renal denervation; nd, no data; SBP, systolic blood pressure.

* The trial was also powered for this efficacy endpoint.

** Not prospectively powered. Proof-of-concept trials. There were no powered endpoints in the trials.

### Sample size recalculations

Three sham-RCTs were designed to show superiority of renal denervation over sham intervention, two were not prospectively powered, and in one trial the authors do not specify their perspective ( Table 2, Box 2). We were able to replicate the sample size calculations in 3 of the trials ^21^^,^^22^^,^^27^ and in 2 of the studies no power analyses were performed. ^24^^,^^25^ In one study ^19^^,^^20^ the power calculation was made for both the safety and subsequently for the efficacy primary outcome based on historical data and we were not able to replicate these ( Table 2, Box 2, Online Table 1 in the *Extended data* ^43^).

**Table 2.** Sample size assumptions and conditional sample size calculations.

Trials	Details about sample size calculations as reported in the paper			Achieved sample size (active vs. sham (cumulative total sample size))	Recalculated sample size using the reported details (active vs. sham)	Recalculated sample size using the effect size from the meta-analysis (active vs. sham) **	Conditional planning sample size (active vs. sham) ***	Relative increase between achieved and calculated sample size (%)	Observed mean difference (SE) (mmHg)	Cumulative mean difference based on meta-analysis (mmHg)
	Calculated sample size in (active vs. sham)	Planned power	Anticipated mean difference (standard deviation) (mmHg) *							
SYMPLICITY HTN-3 ^19^^,^^20^	316 *vs.* 158	95%	−5 (25)	364 *vs.* 171 (535)	975 *vs.* 488 ****	na	na	11	−1.96 (1.54)	−1.96
Desch S., *et al*. ^21^	29 *vs.* 29	80%	−6 (8)	35 *vs.* 36(606)	29 *vs.* 29	263 *vs.* 263	1050 *vs.* 1050	18	−3.50 (2.55)	−2.37
ReSET ^22^	28 *vs.* 28	80%	−10 (13)	36 *vs.* 33 (675)	28 *vs.* 28	474 *vs.* 474	260 *vs.* 260	19	−1.10 (3.53)	−2.22
SPYRAL HTN-OFF MED ^23^^,^^24^	na	na	na	38 *vs.* 42 (755)	na	na	250 *vs.* 250	na	−5.00 (2.51)	−2.75
SPYRAL HTN-ON MED ^23^^,^^25^	na	na	na	38 *vs.* 42 (835)	na	na	0	na	−7.40 (2.63)	−3.45
RADIANCE-HTN SOLO ^26^^,^^27^	64 *vs.* 64	80%	−6 (12)	74 *vs.* 72 (981)	64 *vs.* 64	190 *vs.* 190	0	12	−1.60 (2.04)	−3.08

Abbreviations: na, not applicable; SE, standard error; UCI, upper confidence interval; LCI, lower confidence interval.

* Assumed difference (mean and standard deviation) between the two groups of interventions for the respective primary efficacy outcome in each trial.

** Calculated based at each stage on the previous meta-analysis for mean difference, standard deviation of the one considered by the investigators in individual trials and assumed 80% power.

*** Calculated based at each stage on the previous meta-analysis for mean difference, standard deviation of 10 (the minimum observed in any arm) and assumed 80% power.

**** We were not able to recalculate the sample size calculations of the specific trial even after contacting the principal investigator of the trial.

Box 2. **Power calculations as reported in individual sham-RCTs.**

**Table:** 

Trial	Power calculation description
SYMPLICITY HTN-3 ^19^^,^^20^	“ … In agreement with the Food and Drug Administration, the superiority of denervation over the sham procedure was established by a margin of 5 mmHg for the primary efficacy end point and by a margin of 2 mmHg for the secondary efficacy end point. The superiority margin of 5 mmHg for the primary efficacy end point was considered a clinically meaningful blood-pressure reduction on the basis of the observed decreases in cardiovascular morbidity with small reductions in systolic blood pressure (2 to 5 mmHg) with pharmacologic therapy. The detailed power and sample-size calculations have been published previously … ”, “… Regarding the primary effectiveness end point, a reduction in office-based SBP of ≥5 mmHg is considered a clinically meaningful improvement. Specifically, a 5-mm Hg reduction in SBP has been associated with a 14% decrease in stroke, a 9% decline in cardiovascular disease, and 7% reduction in mortality. Assuming a true difference between treatment means of 15mmHg with a 25 mmHg standard deviation of SBP change per group, a sample size of 316 treatment and 158 control subjects provides 95% statistical power to demonstrate a >5-mm Hg difference between treatment groups at a 1-sided alpha level 0.025. …”
Desch S., *et al*. ^21^	“… Sample size was calculated for the between-group comparison with regard to the primary end point. At the time of trial planning, previous data to guide calculation were scarce. The only available randomized trial of RSD in resistant hypertension (Symplicity HTN-2) compared RSD against no-sham control in patients with resistant hypertension and severely elevated BP. ABPM recordings were available for a subgroup of patients: the mean reduction in 24-hour systolic BP at 6 months was 11±15 mmHg in patients assigned to RSD and 3±19 mmHg in control patients (for a net difference of 8 mmHg between groups). For the current trial, we assumed a less pronounced effect of RSD on BP in light of inclusion of patients with only mildly elevated BP. We speculated that RSD would lead to a difference of at least 6 mmHg between groups with regard to the primary end point (75% of the treatment effect observed in Symplicity HTN-2). We assumed a lower SD of systolic BP values based on a more homogeneous population compared with Symplicity HTN-2. Based on data from a previous trial in mildly hypertensive patients, the presumed SD was set at 8 mmHg for both groups. Thus, 29 patients per treatment arm needed to be analyzed to reject the null hypothesis of equal means between the 2 groups to provide a statistical power of 80% (2-sided test, α=0.05). To account for potential dropouts or nonanalyzable ABPM recordings, an additional 20% of patients were randomized in each arm. Sample size was calculated using nQuery Advisor 7.0 (Statistical Solutions, Saugus, MA) …”
ReSET ^22^	“… The ReSET trial was initiated before the HTN3 trial. Therefore, according to ABPM data from the HTN2 trial and according to our own pilot data, we hypothesized a between-group difference on the primary endpoint of 10mmHg (daytime systolic ABPM after 3 months). Expecting a SD of approximate 13mmHg on ABPM (own data), we calculated a minimum sample size of 28 patients in each group, beta value 0.8 and alpha value 0.05. Analysis was planned according to the intention-to-treat principle (meaning from the time of randomization), and we therefore decided to randomize a total of 70 patients … ”
SPYRAL HTN-OFF MED ^23^^,^^24^	“… The current proof-of-concept trial was designed in collaboration with, and approved by, the US Food and Drug Administration (FDA) with consideration of the recommendations in the 2014 Scientific Statement by the American Society of Hypertension, which suggested a phase 2-type trial in a small group of patients. The protocol allowed up to 120 patients to be randomly assigned with prospectively planned interim analyses after 40, 60, 80, or 100 patients had completed the 3-month follow-up. The purpose of each interim analysis was to ascertain whether there was an adequate treatment effect with a sufficient reduction in variability of the blood pressure measurements to allow design of a larger, pivotal trial. All patients enrolled after this decision point will be included in the pivotal dataset, as discussed with the FDA, and thus this report represents the primary results of the SPYRAL HTN-OFF MED trial. There were no powered endpoints in the trial. To do a properly powered randomised trial assuming a 5 mmHg SBP reduction with a standard deviation of 12, it was established that 246 patients would be required. Because of the unsatisfactory outcome of the SYMPLICITY HTN-3 trial, we decided to proceed with a smaller, proof-of-concept trial that would minimise exposure of patients to an interventional procedure and provide sufficient evidence to move forward with a larger, powered trial. Statistical analyses were done according to the intention-to-treat principle. …”
SPYRAL HTN-ON MED ^23^^,^^25^	“… The protocol allowed up to 110 patients to be randomly assigned with prospectively planned interim analyses after 40, 60, and 80 patients completed 3 months follow up, respectively. Because the current study prespecified that patients should be maintained on the same medication regimen through 6 months follow-up, analysis of the 80 patient cohort was then performed to assess the pattern and progression of blood pressure change over time. The purpose of each interim analysis was to confirm the safety of the procedure and determine if the blood pressure lowering effect of renal denervation was sufficient to support design of future trials. There are no powered endpoints in the trial. Statistical analyses were done based on the intention-to-treat principle. …”
RADIANCE-HTN SOLO ^26^^,^^27^	“… Assuming a 6 mmHg difference in change in daytime ambulatory systolic blood pressure at 2 months between the renal denervation and the sham groups,17 a common SD of 12 mmHg, 1:1 randomisation, and a two-sided type 1 error rate of 5%, a sample size of 128 evaluable patients would yield 80% power. To account for up to 10% missing data on the primary endpoint, we planned to randomise a total 146 patients in the study. …”

**Figure 1.**
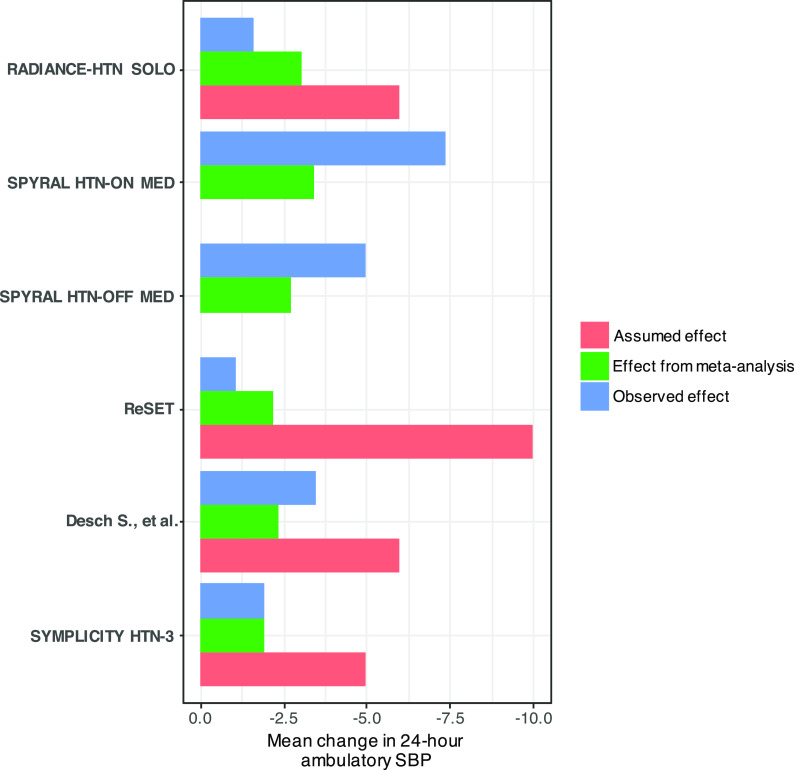
Plot of assumed and observed mean differences in each individual trial, and the cumulative mean difference derived at each step of the cumulative meta-analysis using fixed-effect. Two of the trials (SPYRAL HTN-OFF MED and SPYRAL HTN-ON MED) were designed as proof-of-concept trials. Therefore, they were not prospectively powered and assumed effects are not provided. Abbreviation: SBP, systolic blood pressure.

The achieved sample size was in all cases larger than that calculated and the relative difference was between 11% to 19% ( Table 2). The anticipated mean differences used in sample size calculations by the study authors were larger than those which were actually observed in the trials, or in the trials published before ( Figure 1). Consequently, each study could not detect any important differences between the active and sham interventions. This can be attributed to the over-optimistic effect considered in the sample size calculations to be able to conform with the available sample of patients for recruitment (‘reverse engineered’ sample size calculation).

### Standard and sequential meta-analysis

**Figure 2.**
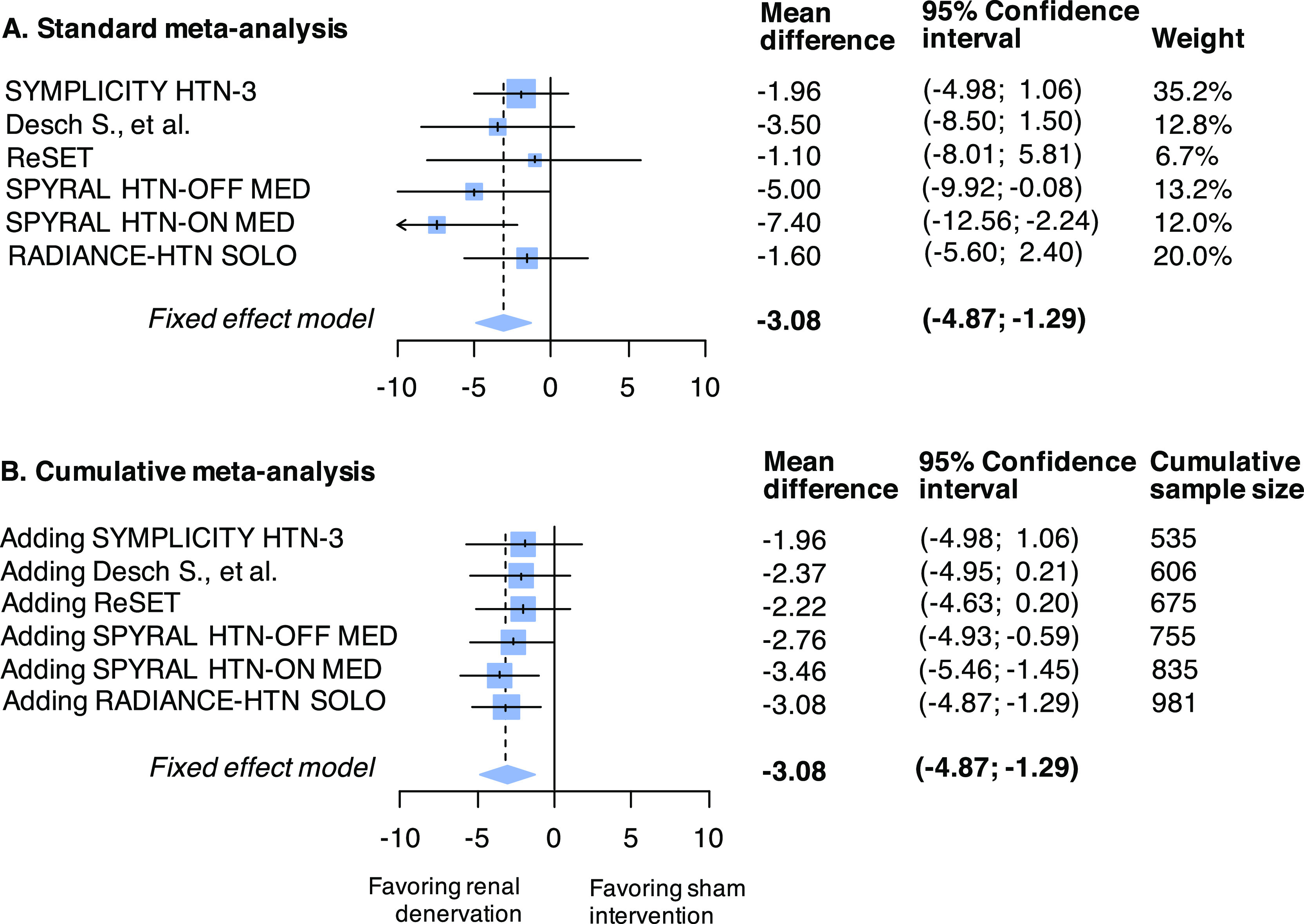
Standard (panel A) and cumulative (panel B) fixed-effect meta-analysis of sham-RCTs comparing renal sympathetic denervation to sham intervention for the outcome of mean change from baseline to follow-up in 24-hour ambulatory systolic blood pressure (mmHg). Abbreviation: RCT, randomized controlled trial.

**Figure 3.**
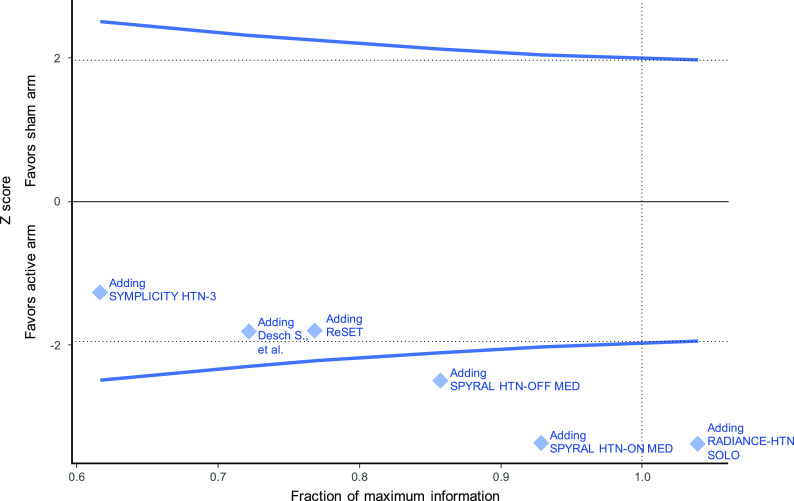
Hypothetical prospectively planned sequential fixed effect meta-analysis framework (type I error=5%, power=90%).

The standard meta-analysis forest plot illustrates the individual results of each trial and its contribution (weight) to the summary effect ( Figure 2 panel A); the cumulative meta-analysis plot shows how the evidence evolved over time ( Figure 2 panel B). Data used are available in Online Table 2 (see underlying *data* ^43^). The estimated heterogeneity variance was zero. If a meta-analysis was conducted immediately after the publication of the fourth study (when 755 patients had been randomized in total), the summary mean difference favoring the active intervention would have been found to be -2.76 (95%CI -4.93 to -0.59). Even after accounting for the sequential nature of the data accumulation, the addition of the fourth study would provide evidence against the null hypothesis ( Figure 3). The final time point is therefore the time of publication of the fourth study (in 2017). The total sample size randomized thereafter (in the fifth and sixth trials) could be considered redundant (226 study participants in total, of which 114 randomized to sham).

### Estimation of the sample size using conditional planning

The sample size of each future study calculated based on conditional power of meta-analysis is presented in Table 2. The summary effect of the meta-analysis after each study was included was much smaller than the anticipated effect used by the authors in their sample size calculations ( Figure 2). Consequently, sample size calculations using the meta-analysis mean difference results to substantially larger calculated sample size compared to that calculated by the trialists ( Table 2).

The large sample sizes calculated with conditional power compared to that calculated by the trials is explained by the fact that the trialists chose unrealistically large anticipated mean differences. If studies had been planned prospectively, the third study would have needed 260 participants per arm and the synthesis of the first three studies would have been enough to reject the null hypothesis. The total sample size from the three trials would have been 1126 (the achieved sample size from the first two trials and the estimated using conditional power from the third trial), while the total achieved sample size in the published studies is 981. Τhis means that the sample size with conditional planning under this scenario is larger than the total observed in the studies ( Figures 1 and 2, Table 2).

## Discussion

Critical review of the available evidence in terms of systematic reviews and meta-analyses of RCTs can provide an in-depth summary of available evidence on a specific topic and contribute in the planning of future research agenda in two ways: by identifying gaps in knowledge on which efforts should be focused, and by contributing to the conditional planning of a future trial based on the relevant existing evidence. ^9^^,^^28^^–^^30^ For the latter, both, pairwise and network meta-analyses, have been proposed as appropriate tools. ^3^^–^^5^^,^^31^ Here, in a retrospectively designed scenario of the particular setting of sham-RCTs, we demonstrated how sequential meta-analysis and conditional planning of a future trial can provide an alternative strategy to the practice of conducting many small, underpowered RCTs with unrealistically large assumed expected treatment differences. Through sequential meta-analysis of sham-controlled trials, investigators can achieve conclusive findings earlier than individual small-scale trials and hence avoid exposing patients to sham-related risks. However, as we illustrated in our example, conditional planning of a future sham-RCT poses important challenges, since invasive procedures may improve over time and the intervention effect is expected to increase in new studies which violates the underlying assumptions.

Systematic reviews of sham-RCTs constitute an ideal setting for considering existing evidence when planning new studies as it is even more imperative to prevent exposure of patients to risks related to the sham intervention. However, conditional planning might in theory result to recommendations of very small trials, which would be associated with great within-study variance and not be standalone experiments. Setting a minimum sample size for a future trial designed using conditional planning would be a potential remedy for such a situation. The dataset of trials we used, which has been previously extensively synthesized in meta-analyses, ^13^ was no exception to the practice of setting large expected differences. The exaggerated power calculations were also reflected by the fact that the achieved sample size was always larger than the calculated. Moreover, individual trials in the early phase resulted in conflicting findings compared to subsequent trials, although statistical heterogeneity was estimated at zero. ^32^ Differences among the trials were attributed to variability in sample sizes, study design (i.e. proof-of-concept trials), blinding of outcomes assessors, patient characteristics, modification of procedural technique and ablation catheters over time, physicians’ experience, medical treatment protocols, and outcome adjudication methods which may yield differences not only among the trials but even in the same trial. ^13^^,^^32^ Nevetheless, the resulted sample sizes based on conditional planning were much larger than those used in individual trials. This can be also attributed to the overoptimistic expected effect sizes in individual trials and to a small trend of increase in the intervention effect over time, possibly because of a learning curve effect in performing the specific procedure.

Clinical research is characterized by sequential flow. New studies are built on the knowledge of the previous ones by using either prior information in making the decision to conduct a new trial or meta-analysis of existing evidence to design the subsequent trial. Even though both approaches have been established under different conditions, concerns have been raised regarding potential sources of biases due to the sequential design, particularly when a clinically relevant effect is ignored in sample size calculations. ^33^ In this scenario, appropriate specification of clinically relevant effects is an important aspect in planning future trials to avoid unrealistic expectations. Along these lines, previous evaluations have shown the appropriateness of conditional planning under different scenarios of inconclusive meta-analysis (confidence interval of the summary effect includes effect sizes with different implications). ^3^ Further development and establishment of evidence-base sample size calculation approaches that would move away from the principles of statistical significance would be an important step forward in the field.

Conditional planning in a frequentist or Bayesian framework can be applied for planning future research agenda. ^4^^,^^5^^,^^34^^–^^37^ Nowadays, clinical trials are becoming costly and time consuming; whereas consideration of such approaches in planning future trials can potentially overcome obvious challenges (i.e. lower recruitment rates than expected or limited funding sources), better prioritize research agenda and subsequently mitigate the growing problem of wasteful research efforts in the biomedical field. ^9^^,^^30^^,^^38^^,^^39^ It is of obvious importance to better design the required future single study or studies, in order to maximize their efficiency and potentially provide the information needed to make informed decisions in clinical effectiveness research. It could be that a small-scale study is needed to confirm previous findings or alternatively new studies may be deemed unnecessary in a scenario where the existing evidence suggests a small effect size which is unlikely to subsequently change. However, particular attention should be paid on the required assumptions of the method before embarking on applying conditional planning of new trials ( Box 1).

### Limitations

Our evaluation has several limitations. First, we chose an example of relatively limited number of available trials with small sample sizes and special design (sham-RCTs with two of them serving as proof-of-concept studies). A comprehensive simulation study would be a more appropriate tool to investigate the performance and robustness of the method under a variety of settings. Even though our example can be representative of the size of the available sham-RCTs in any medical field, the small number of studies might have resulted in clinical heterogeneity not manifesting in the data as statistical heterogeneity. In a real application, imputing a value for heterogeneity, informed for example by empirical predictive distributions, ^40^^,^^41^ and performing random-effects would be a reasonable model choice. Such an approach would be less reasonable in a retrospective application of the methods and would mitigate the comparability between conventional and evidence-based sample size calculations. Second, sequential methods have inherited limitations since they have been mainly built on the principal of statistical significance and do not differentiate between clinically relevant and non-relevant effects. Along these lines, the Cochrane Handbook authors underline the methodological limitations that arise from sequential methods. ^42^ Third, the applied method of conditional planning is based on aggregated findings of completed trials. However, investigators may need to adapt a trial’s design (i.e. sample size re-calculations) after its launch. These interim findings could potentially provide important insights for the planning of future trials, but available statistical approaches cannot safely consider this information. Finally, we applied a retrospective analysis while aiming to illustrate the process in a hypothetical prospective framework. In an actual application, the process should be planned and undertaken prospectively by a collaborative panel including clinicians, decision makers, methodologists and patient representatives.

## Conclusions

Sequential meta-analysis of sham-controlled trials can help answering the research question earlier and avoid unnecessarily exposing patients to sham-related risks. However, conditional planning of new sham-RCTs poses important challenges. As many surgical/minimally invasive procedures improve over time, the intervention effect is expected to increase in new studies and this violates the underlying assumptions. Unless this expected change is accounted for, conditional planning will not improve the design of sham-RCTs.

## Data availability

### Underlying data

Zenodo: Estimating the sample size of sham-controlled randomized controlled trials using existing evidence. https://doi.org/10.5281/zenodo.5865523. ^43^

This project contains the following underlying data:

- Online Table 2: Mean changes in each group of intervention and the difference between the groups for the efficacy outcome of 24-hour ambulatory systolic blood pressure as given in individual trials

### Extended data

Zenodo: Estimating the sample size of sham-controlled randomized controlled trials using existing evidence. https://doi.org/10.5281/zenodo.5865523. ^43^

This project contains the following extended data:

- Online Box 1: Medline and CENTRAL search algorithm
- Online Figure: Study selection flowchart.
- Online Table 1: Sample-size recalculations in individual sham RCTs in Stata.

Data are available under the terms of the Creative Commons Attribution 4.0 International license (CC-BY 4.0).
