# Supplementary material for: Estimating the sample size of sham-controlled randomized controlled trials using existing evidence
Source: F1000Res. 2022 Nov 7;11:85. Originally published 2022 Jan 24. [Version 2] doi: 10.12688/f1000research.108554.2 (PMC9669514; doi:10.12688/f1000research.108554.2)
Supplement: Peer review history

# Peer review: Reviewer response for version 2

**DOI:** 10.5256/f1000research.140665.r155090

**Published:** 2022-11-16

**Authors:** Waldemar Siemens

**Affiliations:** 1 Institute for Evidence in Medicine, Medical Center - University of Freiburg, Faculty of Medicine, Freiburg, Germany; 2 Cochrane Germany, Cochrane Germany Foundation, Freiburg, Germany

The authors revised the paper and addressed all comments thoroughly. Congratulations to this work.

Is the rationale for developing the new method (or application) clearly explained?

Yes

Is the description of the method technically sound?

Yes

Are the conclusions about the method and its performance adequately supported by the findings presented in the article?

Yes

If any results are presented, are all the source data underlying the results available to ensure full reproducibility?

Partly

Are sufficient details provided to allow replication of the method development and its use by others?

Partly

Reviewer Expertise:

Meta-research, meta-analysis, living systematic review

I confirm that I have read this submission and believe that I have an appropriate level of expertise to confirm that it is of an acceptable scientific standard.

# Peer review: Reviewer response for version 2

**DOI:** 10.5256/f1000research.140665.r155091

**Published:** 2022-11-07

**Authors:** Thomas Karagiannis

**Affiliations:** 1 Clinical Research and Evidence-Based Medicine Unit, Second Medical Department, Aristotle University of Thessaloniki, Thessaloniki, Greece; 2 Diabetes Centre, Second Medical Department, Aristotle University of Thessaloniki, Thessaloniki, Greece

The authors have adequately addressed my comments. I have no more comments to add.

Is the rationale for developing the new method (or application) clearly explained?

Yes

Is the description of the method technically sound?

Yes

Are the conclusions about the method and its performance adequately supported by the findings presented in the article?

Yes

If any results are presented, are all the source data underlying the results available to ensure full reproducibility?

Yes

Are sufficient details provided to allow replication of the method development and its use by others?

Yes

Reviewer Expertise:

Evidence synthesis, Systematic reviews, Diabetes Mellitus

I confirm that I have read this submission and believe that I have an appropriate level of expertise to confirm that it is of an acceptable scientific standard.

# Peer review: Reviewer response for version 1

**DOI:** 10.5256/f1000research.119949.r150812

**Published:** 2022-10-17

**Authors:** Waldemar Siemens

**Affiliations:** 1 Institute for Evidence in Medicine, Medical Center - University of Freiburg, Faculty of Medicine, Freiburg, Germany; 2 Cochrane Germany, Cochrane Germany Foundation, Freiburg, Germany

The authors present an empirical example comparing renal sympathetic denervation to sham intervention and provide data and calculations on power, cumulative meta-analysis, and sequential meta-analysis aiming to present the idea of conditional power. Many important points are well addressed and methodological aspects are well connected to the clinical relevance of the methods. I have some major and minor questions and encourage the authors to comment to improve the manuscript.

**Major comments:**

 Limitations: “performing random-effects would be a reasonable model choice” - Why have you worked with the FE model then?

 If conditional power would say that only a “small trial” is needed: Would this be a problem regarding the sampling error? Should trials have a minimum size to avoid sampling error? As the trial gets smaller, the problem of chance increases, or? Could you comment on that and reflect you approach (conditional power)?

 This is an empirical example. Could a reasonable simulation study add value? If yes, how could it look like? 

**Minor comments:**

*Abstract:*

 You could add more numeric results in the results of you abstract if appropriate.  

*Background:*

 “This typically leads to smaller required sample sizes compared to that obtained using

 the conventional approach.” - I wonder if a disadvantage of this approach is that the sampling error increases with a small sample size in a trial. Could you comment on that?

 “Among others it is assumed that the true underlying effect size (which we assume is unbiasedly estimated by the summary effect) should not change over time. This is rather unlikely to happen in sham-RCTs as the learning curve applies to most surgical/minimally invasive interventions and studies of their efficacy show larger effects over time. Hence, the conditional power approach is both promising and challenging to be applied in this context.” -  Does the fixed-effect (FE) model makes sense in this context? Clinical trials vary in their PICO by nature, which makes it hard to assume the FE model.

 “As heterogeneity was low in this setting, we performed fixed effect meta-analyses.” - Shouldn’t that be a choice based on the homogeneity of the trials according to the PICO scheme? 

*Box 1:*

 “The variability of the true treatment effect across trials should be low. Otherwise, even the planning of huge trials will not result in the anticipated conditional power.”  - This again puts the FE model into question, or? Should the conditional power calculations be based the random-effects model?

*Results:*

 Table 1 / meta-analysis: Is it appropriate to pool “24-hour ambulatory SBP” with “Daytime ambulatory SBP”? 

 Link for “Online Table 2”: Only “Table 2” is a link and leads to Table 2 in the manuscript, not to Online Table 2. Please check all link in the manuscript.

 “heterogeneity variance” (p. 5) - Do you mean the between-study variance Tau^2? Please be more precise. 

 “The total sample size randomized thereafter (in the fifth and sixth trials) could be considered redundant (226 study participants in total, of which 114 randomized to sham).” - I think this should be one key message of the paper exactly with this wording, i.e., how many patients would not receive sham. It adds weight for a clinically meaningful understanding of the methods presented. 

 “The large sample sizes calculated with conditional power compared to that calculated by the trials is explained by the fact that the trialists chose unrealistically large anticipated mean differences.” - Sometimes you already interpret your results in the discussion section. Please move the explaining sentences rather to the discussion. 

 “Figure 3. Hypothetical prospectively planned sequential fixed effect meta-analysis framework (type I error=5%, power=90%).” - I suggest adding more sentences for explaining Figure 3. Not everyone is familiar with sequential meta-analysis so it might be important to help readers understand the idea of it. 

*Discussion:*

 Feasibility of large trials when trial authors would consider conditional power. 

 Stop when rejecting the null hypothesis is defined as the “final time point”, correct? How does this fit to clinical relevance of results? One could argue to stop if a threshold of irrelevance is not anymore included by the 95% CI for example. 

 Have prediction intervals a role in cumulative meta-analysis and sequential meta-analysis to describe heterogeneity?

 Is the R code available? You may add it to zenodo. 

 “Unless this is accounted for, conditional planning will not improve the design of sham-RCTs.” - Could you explicitly say what you mean by “this” to avoid misunderstandings in the conclusion? Somehow I find it hard to follow, maybe also because you word your statement with a negation. Please consider rewriting it.

Is the rationale for developing the new method (or application) clearly explained?

Yes

Is the description of the method technically sound?

Yes

Are the conclusions about the method and its performance adequately supported by the findings presented in the article?

Yes

If any results are presented, are all the source data underlying the results available to ensure full reproducibility?

Partly

Are sufficient details provided to allow replication of the method development and its use by others?

Partly

Reviewer Expertise:

Meta-research, meta-analysis, living systematic review

I confirm that I have read this submission and believe that I have an appropriate level of expertise to confirm that it is of an acceptable scientific standard, however I have significant reservations, as outlined above.

# Response

**Published:** 2022-11-02

**Authors:** Georgios Siontis

**Affiliations:** Department of Cardiology, University Hospital of Bern, Inselspital, Bern, Switzerland, Switzerland

We were pleased to receive the comments of the Reviewer. We are grateful for the very insightful comments that helped us to improve our work further. In the revised version of our manuscript we have addressed all of the suggestions/comments made by the Reviewer. In more detail:

**Reviewer 2:**

**The authors present an empirical example comparing renal sympathetic denervation to sham intervention and provide data and calculations on power, cumulative meta-analysis, and sequential meta-analysis aiming to present the idea of conditional power. Many important points are well addressed and methodological aspects are well connected to the clinical relevance of the methods. I have some major and minor questions and encourage the authors to comment to improve the manuscript.**

**Reply: **Thank you for your feedback.

**Major comments:**

**Limitations: “performing random-effects would be a reasonable model choice” - Why**

**have you worked with the FE model then?**

**Reply: **We thank the reviewer for this comment. In fact, our intention was to employ a random-effects meta-analysis model. As the between-study variance (r ^2^) estimation was 0, this was equivalent to fixed-effect. We realize that this was not clearly stated, and we thus now have rephrased to clarify our approach. In particular, we write in the ‘Standard and sequential meta-analysis’ section: *“We intended to perform random-effects meta-analysis, but as between-study variance* *(r ^2^* *)* *was estimated at 0 in this setting, our calculations are identical to those from a fixed effect meta-analysis.”*. We did not change the term “fixed-effect” in the abstract and the figure legends.

 An alternative strategy would be to inform heterogeneity from empirical distributions (our references 40 and 41). However, such an approach would be appropriate in a prospective application of sequential meta-analysis and conditional planning, rather than a retrospective application, like the one presented in this paper. This is because in a prospective application, estimation of heterogeneity in the first stages of the reviews would be suboptimal. Moreover, imputing a value for heterogeneity would make results from “conventional” and “evidence-based” sample size calculations non-comparable.

 We already wrote in the Discussion: *“* *In a real application, imputing a value for heterogeneity, informed for example by empirical predictive distributions* *[40, 41]* *, and performing random-effects would be a reasonable model choice”*.  And we now added: *“Such an approach would be less reasonable in a retrospective application of the methods and would mitigate the comparability between conventional and evidence-based sample size calculations.”*

**If conditional power would say that only a “small trial” is needed: Would this be a problem regarding the sampling error? Should trials have a minimum size to avoid sampling error? As the trial gets smaller, the problem of chance increases, or? Could you comment on that and reflect you approach (conditional power)?**

**Reply: **This is a topic that would benefit from further investigation. On one hand, a small trial should be a desirable outcome of the conditional planning method, on the other hand it would indeed be associated with greater within-study variation. Although this potentially large within-study variation is in theory incorporated in the conditional planning method (saying for example that an addition of such a study would render conclusive the updated meta-analysis effect, which is our goal), it would probably question the requirement of such individual study to be a standalone experiment. We added in the Discussion: *“However, conditional planning might in theory result to recommendations of very small trials, which would be associated with great within-study variance and not be standalone experiments. Setting a minimum sample size for a future trial designed using conditional planning would be a potential remedy for such a situation.”*.

**This is an empirical example. Could a reasonable simulation study add value? If yes, how could it look like?**

**Reply: **We thank the reviewer for this interesting comment. We do believe that a simulation study could contribute on the evaluation of the robustness of the method, and this could be a follow-up project. In such a simulation study, one should construct scenarios reflecting various assumptions being and not being met. We added under “Limitations”: *“A comprehensive simulation study would be a more appropriate tool to investigate the performance and robustness of the method under a variety of settings.”*

**Minor comments:**

**Abstract:**

**You could add more numeric results in the results of you abstract if appropriate.**

**Reply: **We have added in the Abstract: “ *Conditional planning resulted in much larger sample sizes compared to those in the original trials (relative increase between achieved and calculated sample size ranged between 11-19%),* …”.

****

**Background:**

**“This typically leads to smaller required sample sizes compared to that obtained using**

**the conventional approach.” - I wonder if a disadvantage of this approach is that the sampling error increases with a small sample size in a trial. Could you comment on that?**

**Reply: **Please see our response on your second comment from ‘Major comments’.

**“Among others it is assumed that the true underlying effect size (which we assume is unbiasedly estimated by the summary effect) should not change over time. This is rather unlikely to happen in sham-RCTs as the learning curve applies to most surgical/minimally invasive interventions and studies of their efficacy show larger effects over time. Hence, the conditional power approach is both promising and challenging to be applied in this context.” -  Does the fixed-effect (FE) model makes sense in this context? Clinical trials vary in their PICO by nature, which makes it hard to assume the FE model.**

**Reply: **Please see our response on your first comment from ‘Major comments’. Our intention was to perform a random-effects meta-analysis, but heterogeneity being 0 ended up to a fixed-effect model.

**“As heterogeneity was low in this setting, we performed fixed effect meta-analyses.” - Shouldn’t that be a choice based on the homogeneity of the trials according to the PICO scheme?**

**Reply: **We have now rephrased this sentence to: *“We intended to perform random-effects meta-analysis, but as between-study variance* *(r ^2^* *)* *was estimated at 0 in this setting, our calculations are identical to those from a fixed effect meta-analysis.”*

**Box 1:**

**“The variability of the true treatment effect across trials should be low. Otherwise, even the planning of huge trials will not result in the anticipated conditional power.”  - This again puts the FE model into question, or? Should the conditional power calculations be based the random-effects model?**

**Reply: **Please see our response on your first comment from ‘Major comments’. Furthermore, we acknowledge that heterogeneity might be estimated to be zero due to large within-study variation. We write in the ‘Limitations’ section: *“Even though our example can be representative of the size of the available sham-RCTs in any medical field, the small number of studies might have resulted in clinical heterogeneity not manifesting in the data as statistical heterogeneity.”*.

**Results:**

**Table 1 / meta-analysis: Is it appropriate to pool “24-hour ambulatory SBP” with “Daytime ambulatory SBP”?**

**Reply: **Thank you for mentioning this. Yes, both measurements are highly consistent in changes of similar magnitude for the patient populations recruited in the individual trials. 

**Link for “Online Table 2”: Only “Table 2” is a link and leads to Table 2 in the manuscript, not to Online Table 2. Please check all link in the manuscript.**

**Reply: **Done.

**“heterogeneity variance” (p. 5) - Do you mean the between-study variance Tau^2? Please be more precise.**

**Reply: **The text has been revised as follows: “ *The estimated between-study variance (r ^2^) was zero.*”.

**“The total sample size randomized thereafter (in the fifth and sixth trials) could be considered redundant (226 study participants in total, of which 114 randomized to sham).” - I think this should be one key message of the paper exactly with this wording, i.e., how many patients would not receive sham. It adds weight for a clinically meaningful understanding of the methods presented.**

**Reply: **Thank you for this comment. We now mention in Abstract: “ *Sequential meta-analysis provided firm evidence against the null hypothesis with the synthesis of the first four trials (755 patients, cumulative mean difference -2.75 (95%CI -4.93 to -0.58) favoring the active intervention)), with the fifth and sixth trial to be considered redundant (226 study participants in total, of which 114 randomized to sham).*”

**“The large sample sizes calculated with conditional power compared to that calculated by the trials is explained by the fact that the trialists chose unrealistically large anticipated mean differences.” - Sometimes you already interpret your results in the discussion section. Please move the explaining sentences rather to the discussion.**

**Reply: **The above-mentioned sentence has been moved to the Discussion as suggested.

**“Figure 3. Hypothetical prospectively planned sequential fixed effect meta-analysis framework (type I error=5%, power=90%).” - I suggest adding more sentences for explaining Figure 3. Not everyone is familiar with sequential meta-analysis so it might be important to help readers understand the idea of it.**

**Reply: **Thank you for pointing this issue. We now mention in Methods:  “ *Meta-analyses of medical interventions may result in false positive or false negative results, due to low statistical power when the required number of randomised participants or trials has not been reached. Under this scenario, trial sequential analysis of a meta-analysis may amend these problems by handling a meta-analysis of several RCTs in an analogous manner to interim analysis of a single RCT.*”

**Discussion:**

**Feasibility of large trials when trial authors would consider conditional power.**

**Reply: **We are not sure what the reviewer means with this comment. Is it related to the previous comment about the large within-study variation if small trials are recommended by conditional planning approach? If yes, we refer to our response (and amendments in the paper) on this comment.

**Stop when rejecting the null hypothesis is defined as the “final time point”, correct? How does this fit to clinical relevance of results? One could argue to stop if a threshold of irrelevance is not anymore included by the 95% CI for example.**

**Reply: **A limitation of sequential methods lies on them being based on the principles of statistical significance. We write in the ‘Limitations’: *“Second, sequential methods have inherited limitations since they have been mainly built on the principal of statistical significance and do not differentiate between clinically relevant and non-relevant effects. Along these lines, the Cochrane Handbook authors underline the methodological limitations that arise from sequential methods* *[42]* *.»*

 We could indeed extend the methodology to account for clinically relevant results, but such an approach would require further development and evaluation of its feasibility.

**Have prediction intervals a role in cumulative meta-analysis and sequential meta-analysis to describe heterogeneity?**

**Reply: **Yes, prediction intervals fall naturally within the principles of cumulative meta-analysis, although they have not been used in this way. We refer to the Appendix A2 (a short paragraph) of our methodological paper: *Nikolakopoulou A, Mavridis D, Egger M, Salanti G. Continuously updated network meta-analysis and statistical monitoring for timely decision-making. Statistical Methods in Medical Research. 2016 Jan.*

 We do not think that a discussion of the topic would fit in the current paper but please advise if this was the intention of your comment.

**Is the R code available? You may add it to zenodo.**

**Reply: **Yes. The R code is available upon request.

**“Unless this is accounted for, conditional planning will not improve the design of sham-RCTs.” - Could you explicitly say what you mean by “this” to avoid misunderstandings in the conclusion? Somehow I find it hard to follow, maybe also because you word your statement with a negation. Please consider rewriting it.**

**Reply: **Thank you for your comment. In our conclusive statement, “this” corresponds to the expected increase of the intervention effect in new studies. We have rephrased the conclusive statement as follows: “ *Unless this expected change is accounted for, conditional planning will not improve the design of sham-RCTs.*”

# Peer review: Reviewer response for version 1

**DOI:** 10.5256/f1000research.119949.r129149

**Published:** 2022-04-11

**Authors:** Thomas Karagiannis

**Affiliations:** 1 Clinical Research and Evidence-Based Medicine Unit, Second Medical Department, Aristotle University of Thessaloniki, Thessaloniki, Greece; 2 Diabetes Centre, Second Medical Department, Aristotle University of Thessaloniki, Thessaloniki, Greece

I do not have any particular comments on the methodological aspects of the paper as it appears to be methodologically sound.

 In my opinion, the most important limitation of this methodological study is that the conditional sample size calculation method, as presented by the authors, is based on the principle of statistical significance (i.e. the treatment effect as produced by a meta-analysis of available trials) and not on the rationale/principle of what value is considered clinically relevant (i.e. a conventional approach in which the anticipated treatment effect of an intervention is based on the minimal clinically important difference). I believe that the authors should emphasize this issue in their discussion; I do realize that they make mention to this limitation in the discussion (Limitations section), but I think it should be further elaborated in the context of clinical/practical implications. For example, one might wonder whether an alternative approach combining elements of both the conventional and the conditional approaches might be more reasonable when designing a new trial, i.e. use the minimal clinically important difference in sample calculation and adjust the planned sample size based on previous similar trials in the sense that the cumulative sample of all available trials (be means of a meta-analysis) would be adequately powered to collectively assess the minimal clinically important effect estimate.

 Regardless, I believe that it is noteworthy that this paper highlights various shortcomings/limitations of individual trials when it comes to sample size calculation, such as the use of “reverse engineering” (calculation is based on practical or unspecified considerations resulting in unrealistically large assumed treatment effects which in turn lead to inadequate sample size). I was also interested to see that, based on table 2, the replicated/recalculated sample size (975+488) in SYMPLICITY HTN-3 trial was much larger the sample size originally calculated in the actual trial (316+158), which implicates that sample size calculation in individual studies can still be flawed even when a minimal clinically important difference (not reverse engineering) is used.

 Finally, I noticed in Box 2 that SYMPLICITY HTN-2 trial is mentioned. I am wondering why this trial was not included in the pool of studies.

Is the rationale for developing the new method (or application) clearly explained?

Yes

Is the description of the method technically sound?

Yes

Are the conclusions about the method and its performance adequately supported by the findings presented in the article?

Yes

If any results are presented, are all the source data underlying the results available to ensure full reproducibility?

Yes

Are sufficient details provided to allow replication of the method development and its use by others?

Yes

Reviewer Expertise:

Evidence synthesis, Systematic reviews, Diabetes Mellitus

I confirm that I have read this submission and believe that I have an appropriate level of expertise to confirm that it is of an acceptable scientific standard, however I have significant reservations, as outlined above.

# Response

**Published:** 2022-11-02

**Authors:** Georgios Siontis

**Affiliations:** Department of Cardiology, University Hospital of Bern, Inselspital, Bern, Switzerland, Switzerland

We were pleased to receive the comments of the Reviewer. We are grateful for the very insightful comments that helped us to improve our work further. In the revised version of our manuscript we have addressed all of the suggestions/comments made by the reviewer. In more detail:

**Reviewer 1:**

**I do not have any particular comments on the methodological aspects of the paper as it appears to be methodologically sound.**

**Reply: **Thank you for your feedback.

**In my opinion, the most important limitation of this methodological study is that the conditional sample size calculation method, as presented by the authors, is based on the principle of statistical significance (i.e. the treatment effect as produced by a meta-analysis of available trials) and not on the rationale/principle of what value is considered clinically relevant (i.e. a conventional approach in which the anticipated treatment effect of an intervention is based on the minimal clinically important difference). I believe that the authors should emphasize this issue in their discussion; I do realize that they make mention to this limitation in the discussion (Limitations section), but I think it should be further elaborated in the context of clinical/practical implications. For example, one might wonder whether an alternative approach combining elements of both the conventional and the conditional approaches might be more reasonable when designing a new trial, i.e. use the minimal clinically important difference in sample calculation and adjust the planned sample size based on previous similar trials in the sense that the cumulative sample of all available trials (be means of a meta-analysis) would be adequately powered to collectively assess the minimal clinically important effect estimate.**

**Reply: **We thank the reviewer for this thought-provoking comment. We certainly agree that methodological developments, along with interpretation of findings in clinical applications, should move away from the principle of statistical significance. We argue, however, that conventional sample size calculations are also based on statistical significance, despite making use of a minimal clinically important difference. What is measured (in conventional sample size calculations) is the expected sample size to detect the minimal clinically important difference as statistically significant. Thus, conditional power used in this paper in fact makes the two approaches comparable.

 Alternative evidence-based sample size calculation approaches include planning new studies based on a desired precision of the updated meta-analysis effect. We write in the Discussion: *“Along these line, previous evaluations have shown the appropriateness of conditional planning under different scenarios of inconclusive meta-analysis (confidence interval of the summary effect includes effect sizes with different implications)* *[3]* *.” *And we also added: *“* *Further development and establishment of evidence-base sample size calculation approaches that would move away from the principles of statistical significance would be an important step forward in the field.”.*

**Regardless, I believe that it is noteworthy that this paper highlights various shortcomings/limitations of individual trials when it comes to sample size calculation, such as the use of “reverse engineering” (calculation is based on practical or unspecified considerations resulting in unrealistically large assumed treatment effects which in turn lead to inadequate sample size). I was also interested to see that, based on table 2, the replicated/recalculated sample size (975+488) in SYMPLICITY HTN-3 trial was much larger the sample size originally calculated in the actual trial (316+158), which implicates that sample size calculation in individual studies can still be flawed even when a minimal clinically important difference (not reverse engineering) is used.**

**Reply: **We thank the reviewer for this concrete comment.

**Finally, I noticed in Box 2 that SYMPLICITY HTN-2 trial is mentioned. I am wondering why this trial was not included in the pool of studies.**

**Reply: **Thank you for pointing this. Indeed, SYMPLICITY HTN-2 trial was not included in the current analysis because the control arm was “standard of care” and not a sham intervention.
